# TMEM175 mediates Lysosomal function and participates in neuronal injury induced by cerebral ischemia-reperfusion

**DOI:** 10.1186/s13041-020-00651-z

**Published:** 2020-08-15

**Authors:** Mengling Zhang, Haifeng Lu, Xueshun Xie, Haitao Shen, Xiang Li, Yunhai Zhang, Jiang Wu, Jianqiang Ni, Haiying Li, Gang Chen

**Affiliations:** 1 Department of Neurology, The First Affiliated Hospital of Soochow University, Suzhou, Jiangsu Province China; 2 Department of Neurosurgery & Brain and Nerve Research Laboratory, The First Affiliated Hospital of Soochow University, Suzhou, Jiangsu Province China; 3 Jiangsu Key Laboratory of Medical Optics, Suzhou Institute of Biomedical Engineering and Technology, Chinese Academy of Sciences, Suzhou, China

**Keywords:** Ischemic stroke, Ischemia-reperfusion injury, Neuron, Lysosome, TMEM175

## Abstract

As the main organelles for the clearance of damaged proteins and damaged organelles, the function of lysosomes is crucial for maintaining the intracellular homeostasis of long-lived neurons. A stable acidic environment is essential for lysosomes to perform their functions. TMEM175 has been identified as a new K^+^ channel that is responsible for regulating lysosomal membrane potential and pH stability in neurons. This study aimed to understand the role of TMEM175 in lysosomal function of neurons and neuronal injury following cerebral ischemia-reperfusion (I/R). A middle-cerebral-artery occlusion/reperfusion (MCAO/R) model was established in adult male Sprague-Dawley rats in vivo, and cultured neurons were exposed to oxygen-glucose deprivation/reoxygenation (OGD/R) to mimic ischemia-reperfusion (I/R) injury in vitro. We found that the protein level of TMEM175 decreased after cerebral I/R injury and that TMEM175 overexpression ameliorated MCAO/R-induced brain-cell death and neurobehavioral deficits in vivo. Furthermore, these results were recapitulated in cultured neurons. Acridine orange (AO) staining, as well as LysoSensor Green DND-189, cathepsin-B (CTSB), and cathepsin-D (CTSD) activities, showed that TMEM175 deficiency inhibited the hydrolytic function of lysosomes by affecting lysosomal pH. In contrast, TMEM175 upregulation reversed OGD/R-induced lysosomal dysfunction and impaired mitochondrial accumulation in cultured neurons. TMEM175 deficiency induced by cerebral I/R injury leads to compromised lysosomal pH stability, thus inhibiting the hydrolytic function of lysosomes. Consequently, lysosomal-dependent degradation of damaged mitochondria is suppressed and thereby exacerbates brain damage. Exogenous up-regulation of TMEM175 protein level could reverse the neuronal lysosomal dysfunction after ischemia-reperfusion.

## Introduction

Cerebral ischemia is a form of stroke that causes considerable disability and mortality worldwide, which induces a heavy burden to both society and affected families. A brief lack of sufficient blood supply can result in severe brain damage, and reperfusion following ischemia is thought to contribute to delayed secondary brain damage [1–3].

Reperfusion following focal cerebral ischemia leads to cellular changes, including the accumulation of misfolded proteins and organelle damage; in particular, significant mitochondrial dysfunction occurs, such as mitochondrial permeability-transition-pore opening, mitochondrial morphological damage, Ca2^+^-induced mitochondrial swelling, and the release of mitochondrial cytochrome c into the cytosol [4]. Mitochondria have been implicated as central players in the development of ischemic cell death, both through impairment of their normal roles in generating ATP for neuronal function and as key mediators in cell-death pathways [5]. As a result, mitochondrial turnover via the removal of damaged mitochondria is critical to neuronal survival. Recent studies have shown that mitophagy, a lysosomal degradative pathway, is essential for maintaining neuronal homeostasis via the removal of dysfunctional mitochondria following cerebral ischemia-reperfusion (I/R) injury [6, 7]. Therefore, the hydrolytic function of lysosomes plays a vital role in neuroprotection against ischemic brain injury.

The activities of abundant lysosomal proteases that contribute to the hydrolytic function of lysosomes are inhibited by unstable pH [8]. Transmembrane protein 175 (TMEM175), recently identified from a lysosomal proteome, is a K^+^ channel located in late endosomes and lysosomes [9]. TMEM175 has been shown to regulate lysosomal membrane potential, pH stability, and organelle fusion via potassium conductance across lysosomal and endosomal membranes in neurons [9]. TMEM175 deficiency impairs lysosomal pH stability, lysosome-mediated autophagosomal clearance, and mitochondrial clearance in neurons [10]. Dysfunctional mitophagy resulting from TMEM175 loss-of-function mutations has been implicated in several human CNS diseases, including Alzheimer’s disease [11] and Parkinson’s disease [7]. However, the role of TMEM175 in lysosomal hydrolysis and mitochondrial quality control after brain I/R injury is unclear. In this study, we aimed to understand the roles of TMEM175 in lysosomal function of neurons and neuronal injury following cerebral I/R.

## Materials and methods

### Experimental design

In experiment 1(Supplementary Figure 1), the levels of TMEM175 and LAMP2 in brain tissues of rats after MCAO/R and in primary neurons after OGD/R were measured. Forty-two male SD rats were randomly and equally assigned to seven groups of six rats each, as follows: a sham group; and six experimental groups at 1, 3, 6, 12 24, and 48 h after MCAO/R. At the corresponding time following induction of MCAO/R, all rats were euthanized by chloral hydrate, and their cerebral tissues were collected for subsequent analysis after transcardial perfusion with PBS. Brain tissues of six rats in each group were extracted and frozen at − 80 °C until further analysis via Western blotting. Similarly, primary neurons were divided into eight groups as follows: a control group; and OGD/R treatment groups at 1, 3, 6, 12, 24, 48, and 72 h after OGD/R. At the corresponding times, cellular proteins were extracted for Western blot analysis.

In experiment 2(Supplementary Figure 1), the effect of TMEM175 deficiency on neuronal apoptosis and long-term behavior was assessed. Here, 170 male SD rats were randomly divided into four groups of 34 rats each, as follows: (1) sham group;(2) sham + Over-TMEM175 group, in which all rats were administered a TMEM175 plasmid (3) 24-h MCAO/R group; (4) MCAO/R + vector group, in which all rats were injected with an empty vector in the lateral cerebral ventricle before 48 h of MCAO/R treatment; and (5) MCAO/R + TMEM175-overexpression group, in which all rats were subjected to MCAO/R and administered a TMEM175 plasmid (dissolved in Entranster-in vivo DNA transfection reagent) before 48 h of MCAO/R treatment. Then, the 34 rats in each group except the sham + Over-TMEM175 group were randomly separated into four subgroups via a random-number table. For six rats, total coronal sections containing penumbral brain tissues were harvested and frozen in liquid nitrogen for Western blot analysis. For another six rats, penumbral brain tissues were harvested for Fluoro-Jade C (FJC) staining. Six rats were sacrificed for 2,3,5-Triphenyltetrazolium Chloride (TTC) staining. For behavioral assessments, experimenters were blind to the component of the infusion and the group of rats. Ten rats were trained on the third, fifth, seventh, and fourteen days after surgery in the rotarod test and adhesive-removal test. We trained the last six rats in the Morris Water Maze. For in-vitro experiments, neuronal cultures were randomly divided into the following four groups: (1) control group; (2) control + TMEM175-overexpression group, in which neurons were administered a TMEM175 plasmid (3) 4-h OGD/R group; (4) OGD/R + vector group, in which neurons were transfected with an empty vector before 48 h of OGD/R treatment; and (5) OGD/R + TMEM175-overexpression group, in which neurons were subjected to OGD/R and administered a TMEM175 plasmid (dissolved in Entranster-in vivo DNA transfection reagent). Then, neuronal death was assessed via Hoechst-33,258 nuclear staining and a live/dead viability assay.

In experiment 3(Supplementary Figure 1), Four hours after OGD/R, mitochondrial ROS was assessed via Mito-SOX dye. Furthermore, assessment of ΔΨm was detected via JC-1 dye at the same time point. We also investigated the role of TMEM175 loss-of-function mutations on the hydrolytic function of lysosomes in vitro: AO staining conducted at 2 h, 4 h after OGD/R treatment and LysoSensor Green DND-189 conducted at 4 h after OGD/R treatment were used to detect changes in lysosomal pH. The activities of the major lysosomal proteases, CTSB and CTSD, were measured at 4 h after OGD/R treatment to detect the hydrolytic function of lysosomes.

### Animals

Animal protocols were approved by the Ethics Committee of the First Affiliated Hospital of Soochow University (Jiangsu Province, China). All animal experiments were in accordance with the National Institute of Health (Bethesda, MD, USA) guidelines for the care and use of experimental animals. Adult male Sprague-Dawley (SD) rats (300–350 g) were purchased from the Animal Center of the Chinese Academy of Sciences (Shanghai, China). Rats were maintained in a room with controlled temperature (22 ± 2 °C), relative humidity (55 ± 5%), and a regular light/dark cycle. Food and water were provided ad libitum. We made every effort to reduce the number of animals used and their suffering.

### Establishment of experimental middle-cerebral-artery occlusion/reperfusion (MCAO/R) model in rats

We established a middle-cerebral-artery occlusion/reperfusion model in SD rats, as previously described [12]. Under an operating microscope, the common, internal, and external carotid arteries (CCA, ICA, and ECA, respectively) were exposed through a midline cervical incision. Then, a piece of filament (Xi Nong, Beijing, China) was inserted into the right CCA and advanced along the right ICA until the tip occluded the proximal stem of the middle cerebral artery. After 2 h of ischemia, the filament was withdrawn for reperfusion. Sham controls only had exposed blood vessels through a midline cervical incision.

### Cell cultures

Primary rat cortical neurons were obtained and cultured as described previously [13]. Pregnant SD rats (16–18 days) were used to prepare primary-neuron-enriched cultures. In brief, we first removed the meninges and blood vessels of the brain. The brain tissues were then digested with 0.25% trypsin for 5 min, and the digestion was terminated by washing the tissue three times with PBS. The brain tissue suspension was centrifuged at 500×g for 5 min, and the pellet was then resuspended in neural basal medium (all from GIBCO, Carlsbad, CA, USA). Finally, cells were seeded in 12-well plates and 6-well plates in fresh medium. Afterward, half of the medium was changed every 2 d.

### In-vitro application of oxygen-glucose deprivation/Reoxygenation (OGD/R)

To mimic I/R in vitro, primary neurons were exposed to oxygen-glucose deprivation/reoxygenation (OGD/R), as previously described [14]. Briefly, the cells were maintained in glucose-free medium in a humidified incubator containing 95% N_2_ and 5% CO_2_ at 37 °C for 2 h. For reoxygenation with glucose reintroduction, cells were again cultured in standard medium and placed in an incubator containing 70% N_2_, 25% O_2_, and 5% CO_2_.

### Western blotting

Western blot analysis was performed as previously reported [15, 16]. Briefly, the brain samples of rats or harvested neurons in vitro were mechanically lysed in a RIPA lysate buffer (Beyotime, China). The protein concentrations were measured by the bicinchoninic acid (BCA) method using a specific assay kit (Beyotime, China). The protein samples (30 μg/lane) were loaded onto a 10% SDS-polyacrylamide gel, separated, and then electrophoretically transferred to a polyvinylidene-difluoride (PVDF) membrane (Millipore Corporation, USA). The membrane was blocked with 5% bovine serum albumin (BSA, BioSharp, China) at 25 °C for 1 h. Next, the PVDF membrane was incubated with primary antibodies at 4 °C overnight. The primary antibodies used in this study were as follows: Transmembrane protein 175 (TMEM175) (Proteintech, China, 1:1000 dilution). In addition, β-tubulin (Cell Signaling Technology, USA) served as a loading control. Finally, the PVDF membrane was incubated with an HRP-conjugated secondary antibody (Cell Signaling Technology, USA) at 25 °C for 1 h. Protein bands were visualized using an Enhanced Chemiluminescence (ECL) kit (Beyotime, China), and the relative amounts of proteins were analyzed via ImageJ software (NIH, USA) and normalized to the corresponding control sample. In addition, phosphorylation levels were assessed as the ratio of phosphoprotein to total protein.

### 2,3,5-Triphenyltetrazolium chloride (TTC) staining

TTC staining was used to detect the infarct volume and was performed as previously reported [17]. Brains were quickly removed, frozen at − 20 °C for 10 min, and sectioned coronally into 2-mm-thick slices starting from the frontal pole. The olfactory bulb and cerebellum were discarded. Then, the brain slices were immersed in TTC solution (Jiancheng Biotech, Nanjing, China) for 15–30 min at 37 °C. After staining, the brain tissue was differentiated according to the white-colored infarct area and red-purple non-infarct area. Then, these areas of brain tissues were imaged with a digital camera. Cerebral infarct volumes were calculated according to the following formula: (contralateral hemisphere area − (ipsilateral hemisphere area – infarct area)/contralateral hemisphere area) × 100%.

### Fluoro-jade C (FJC) staining

FJC staining was used to evaluate neuronal degeneration. Brain sections were deparaffinized, rehydrated, and incubated in 0.06% K permanganate for 15 min. Then, the brain sections were rinsed in deionized water, immersed in 0.001% FJC working solution (0.1% acetic acid) for 30 min, and dried in an incubator (50–60 °C) for 10 min. The brain sections were cleared in xylene, cover slipped with one drop of mounting medium, and observed under a fluorescent microscope (OLYMPUS BX50/BX-FLA/DP70; Olympus Co., Tokyo, Japan).

### Cell-death/viability assays

Four hours after OGD, cell viability and death were quantitatively evaluated by Hoechst-33,258 nuclear staining (Beyotime, China) and a live/dead cell-viability assay (Invitrogen, USA). For Hoechst staining, the percentage of cells showing nuclear condensation was quantified and expressed as a function of the total number of neurons (cell-death percentage). A live/dead cell-viability assay was performed as previously described, according to the manufacturer’s instructions. In this assay, red dots (fluorescent ethidium homodimer-1) represent dead cells with compromised membranes, and green dots (fluorescent membrane-permeant calcein AM) represent live cells. For cell counting, three random fields were captured per well by a blinded observer. The experiments were repeated on three independent occasions.

### Acridine Orange (AO) staining

AO staining was used to detect changes in lysosomal pH. In brief, neurons were first rinsed with PBS. Then, cells were stained with AO mix (10 μg/ml in PBS) for 25 s. Finally, stained neurons were imaged under a confocal microscope (ZEISS LSM 880, Carl Zeiss AG, Germany) from three separate experiments.

### Cathepsin-B (CTSB) and Cathepsin-D (CTSD) activities

CTSB and CTSD activities were measured using commercial assay kits according to the manufacturer’s protocols (Biovision, USA). The samples were read in a microplate reader with 400-nm excitation and 505-nm emission for CTSB and 328-nm excitation and 460-nm emission for CTSD.

### LysoSensor green DND-189 staining

Lysosomal staining with the fluorescent acidotropic probe, LysoSensor Green DND-189, was performed according to the manufacturer’s recommendations (Invitrogen,USA). LysoSensor Green was then added directly to the medium at a final concentration of 5 mM and incubated for 2 h at 37 °C, with 5% CO2. At the end of the incubation, cells were directly transferred to a fluorescent microscope for imaging.

### Measurement of mitochondrial reactive oxygen species (ROS)

Mitochondrial reactive oxygen species (ROS) in primary cultured neurons were assessed via Mito-Sox staining. Briefly, neurons were cultured in 12-well plates with glass coverslips and were subjected to different treatments. Next, neurons were incubated with 5-μM Mito-Sox (Invitrogen, USA) for 15 min at 37 °C in the dark. Finally, samples were randomly imaged, and their fluorescent intensities were analyzed via ImageJ software from three independent experiments.

### Assessment of mitochondrial membrane potential (ΔΨm)

Primary neurons were stained with the inner mitochondrial-membrane-potential (ΔΨm) reporter dye, JC-1 (BD Bioscience), per the manufacturer’s instructions. In brief, JC-1 was prepared to a final concentration of 1 μM in diluting buffer, and neurons under different conditions were incubated for 20 min. Stained neurons were examined under a fluorescent microscope. The specific characteristics of JC-1 dye are as follows. When ΔΨm is low, JC-1 exists as a monomer, and green fluorescence can be detected. By contrast, when ΔΨm increases, JC-1 assemble into arrays, termed J-aggregates, that exhibit red fluorescence. Therefore, at high ΔΨm, red fluorescence is detected. Fluorescent analysis was carried out by measuring the total fluorescence of the entire oocyte.

### Plasmid transfection in vivo *and Vitro*

GFP-TMEM175 non-fused overexpressed plasmid and TMEM175 overexpressed plasmid were used to detect the efficiency of plasmid transfection and overexpression. An empty vector was used as a negative control for the TMEM175-overexperssion (Over-TMEM175) construct. Transfection in vivo was performed as described previously [18]. Briefly, 5 μg of TMEM175-overexpression plasmid was dissolved in 5 μl of endotoxin-free water. Then, 10 μl of Entranster-in vivo DNA transfection reagent (Engreen Biosystem Co. Lid, 18,688–11-2) was immediately added to 5 μl of plasmid. The solution was mixed for 15 min at room temperature. Finally, 15 μl of Entranster-in vivo-plasmid mixture was injected intracerebroventricularly under the guidance of a stereotaxic apparatus after anesthesia. We established the MCAO/R model at 24 h after this process. The puncture point of the lateral ventricle was located at 1.5-mm posterior, 1.0-mm lateral, and 3.2-mm below the horizontal plane of bregma. Cultured neurons were transfected with these two plasmids using Lipofectamine® 3000 Transfection Reagent (Invitrogen, L3000–015), according to the manufacturer’s instructions. At 48 h after transfection, neurons were treated with OGD/R.

### Morris water maze

We trained rats 22–25 days after surgery in the Morris water maze. The arena was 50-cm high and 180 cm in diameter and was filled with water to a height of 30 cm at 20–22 °C. The platform was placed approximately 2 cm below the water surface. Each trial continued until the rat found the platform and stood on it for 2 s, or until 60 s had elapsed. After each trial, the rat was placed onto the platform to rest for 20 s. Swimming speeds, as well as latencies and path lengths to reach the platform, were measured as previously described [19]. The behavioral analyses were conducted by an investigator blinded to the treatments.

### Rotarod test

An accelerating rotarod test was employed to measure the motor function of rats [20]. The diameter of the rotarod spindle was 10 cm. The speed of the spindle was increased from 4 to 30 rpm in 60 s, after which 30 rpm was maintained for a maximum of 300 s. When the rats lost their balance and fell off the rotarod, it triggered the sensor, and the time was recorded. Through separation by two panels to prevent the rats from detecting each other, three rats were able to run simultaneously. Before surgery, each rat received three training sessions per day for three consecutive days, and the last three records were counted as the baseline. Then, all rats received a test trial on an accelerating rotarod at all testing days after MCAO/R.

### Adhesive-removal test

The adhesive removal test was performed as described previously [20]. Briefly, rats were removed from their home cages and were trained to become familiar with the testing environment. Then, two small pieces of adhesive-backed paper dots were glued to the wrist of each forelimb. The rats were then gently returned to their testing cages. The time required to contact and remove both stimuli from each limb was recorded in five trials per day for three days. If the rats were able to remove the dots within 10 s at the end of training, the rats were included in the experimental group. Then, all rats received a test trial at all testing days after MCAO/R.

### Statistical analysis

All data are expressed as the mean ± standard error of the mean (SEM). GraphPad Prism7(GraphPad Software, San Diego, USA) was used for statistical analysis. Data were analyzed by one-way analyses of variance (ANOVAs) to compare differences among multiple groups, and Mann-Whitney U tests were used to analyze nonparametric data. Before the aforementioned tests, datasets in each group were tested for normality via Kolmogorov-Smirnov tests. *P* < 0.05 was considered statistically significant (Supplementary Table 1).

## Results

### General observations

No significant differences were observed for body weight, temperature, blood gas, blood glucose, or blood routine-examination data among the experimental groups (data not shown). No animals died (0/40 rats) in the sham group, and the mortality rate of rats was 19.3% (33/171 rats) after induction of MCAO/R (Supplementary Table 2).

### TMEM175 protein levels are decreased in penumbral tissue after ischemia/reperfusion injury

**Fig. 1:**
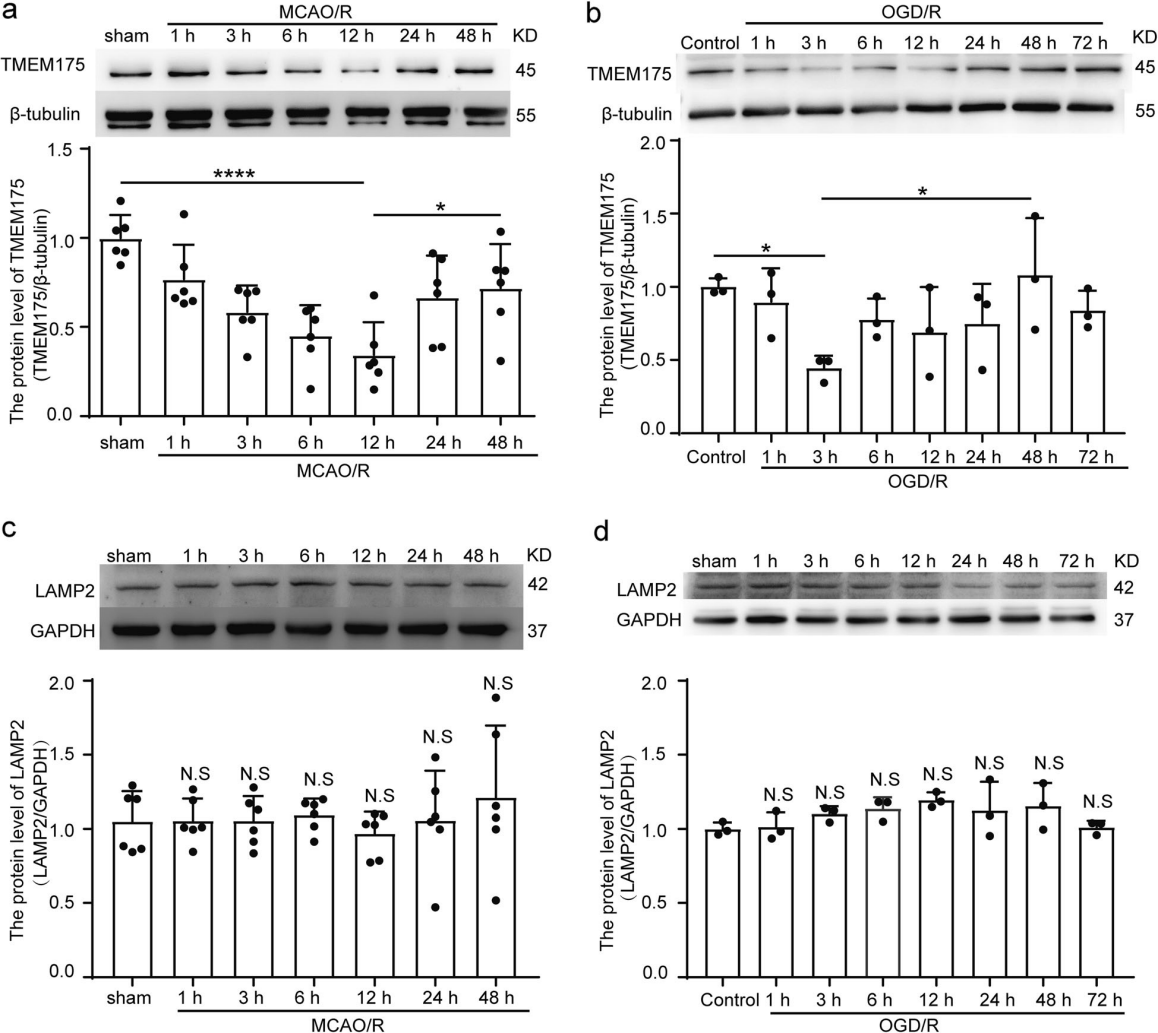
The protein level of TMEM175 in neurons decreases after I/R injury. **a** Western blot analysis and quantification of the protein level of TMEM175 in ischemic penumbra (12 h vs. sham, ****p < 0.0001; 12 h vs. 48 h, *p = 0.0248). **b** Western blot analysis and quantification of the protein level of TMEM175 in cultured neurons exposed to OGD/R (3 h vs. control, *p = 0.0457; 3 h vs. 48 h, *p = 0.0192). **c** Western blot analysis and quantification of the protein level of LAMP2 in ischemic penumbra. **d** Western blot analysis and quantification of the protein level of LAMP2 in cultured neurons exposed to OGD/R. In (**a**–**d**), mean values for the sham group and the control group were normalized to 1.0

To investigate whether the protein level of TMEM175 changes after MCAO/R, we performed MCAO/R in SD rats, and Western blot analysis of protein samples from penumbral tissue was subsequently performed. The protein expression level of TMEM175 is normalized by tubulin expression. Compared with that of the sham group, the protein level of TMEM175 was decreased at 3 h after MCAO/R and dropped to its lowest level at 12 h, after which it gradually rebounded (Fig. 1a). In addition, in order to explore the change of protein level of TMEM175 in primary cultured neurons after OGD/R, we performed Western blot analysis on protein samples. Compared with that of the control group, the protein level of TMEM175 decreased at 3 h after MCAO/R, which is consistent with our results in vivo (Fig. 1b). In order to exclude the possibility that the number of lysosomes decreased after the ischemia/reperfusion injury, the protein level of LAMP2 (lysosomal associated membrane protein 2), a marker for lysosomal and endosomal membranes [21], was investigated in the experiments. We found that both MCAO/R treatment of rats and OGD/R treatment of cultured neurons did not decrease the level of LAMP2. The protein expression level of LAMP2 is normalized by GAPDH expression (Fig. 1c-d). These results suggested that the ischemia/reperfusion injury did not impair the quantity of lysosomes.

### TMEM175 overexpression improves neuronal death and neurobehavioral deficits after MCAO/R in rats

**Fig. 2:**
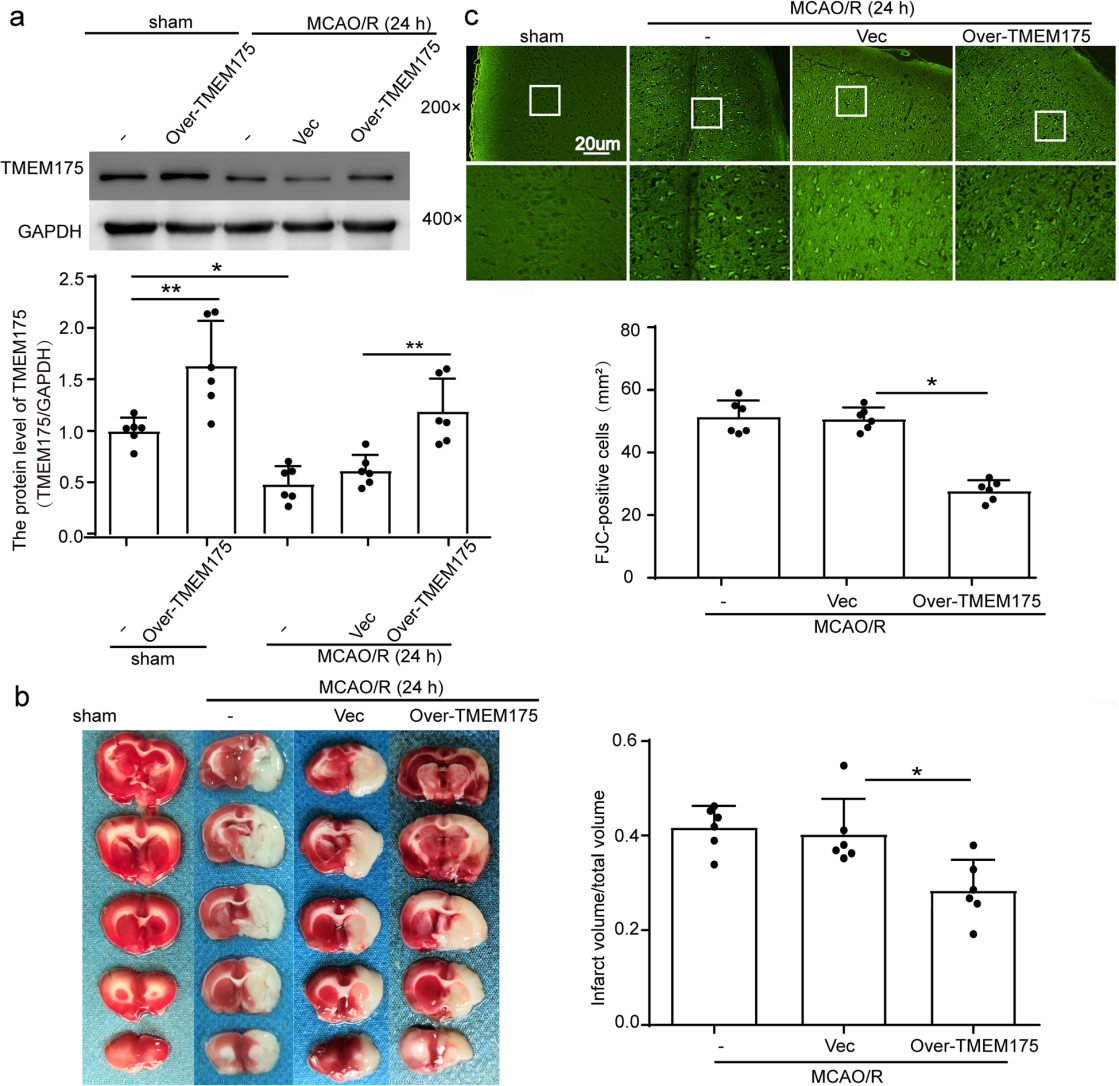
TMEM175 overexpression alleviates brain injury in a rat MCAO/R model. **a** Transfection efficiency of TMEM175 overexpression plasmid in rat brains (MCAO/R vs. sham, *p = 0.0215; MCAO/R + Vector vs. MCAO/R + Over-TMEM175, **p = 0.0091; sham vs. sham + Over-TMEM175, **p = 0.0034). Mean values for the sham group were normalized to 1.0. **b** TTC staining. (Vector vs. Over-TMEM175, *p = 0.0126). **c** FJC staining showing the effects of TMEM175 intervention on neuronal degeneration (Vector vs. Over-TMEM175, ****p < 0.0001)

**Fig. 3:**
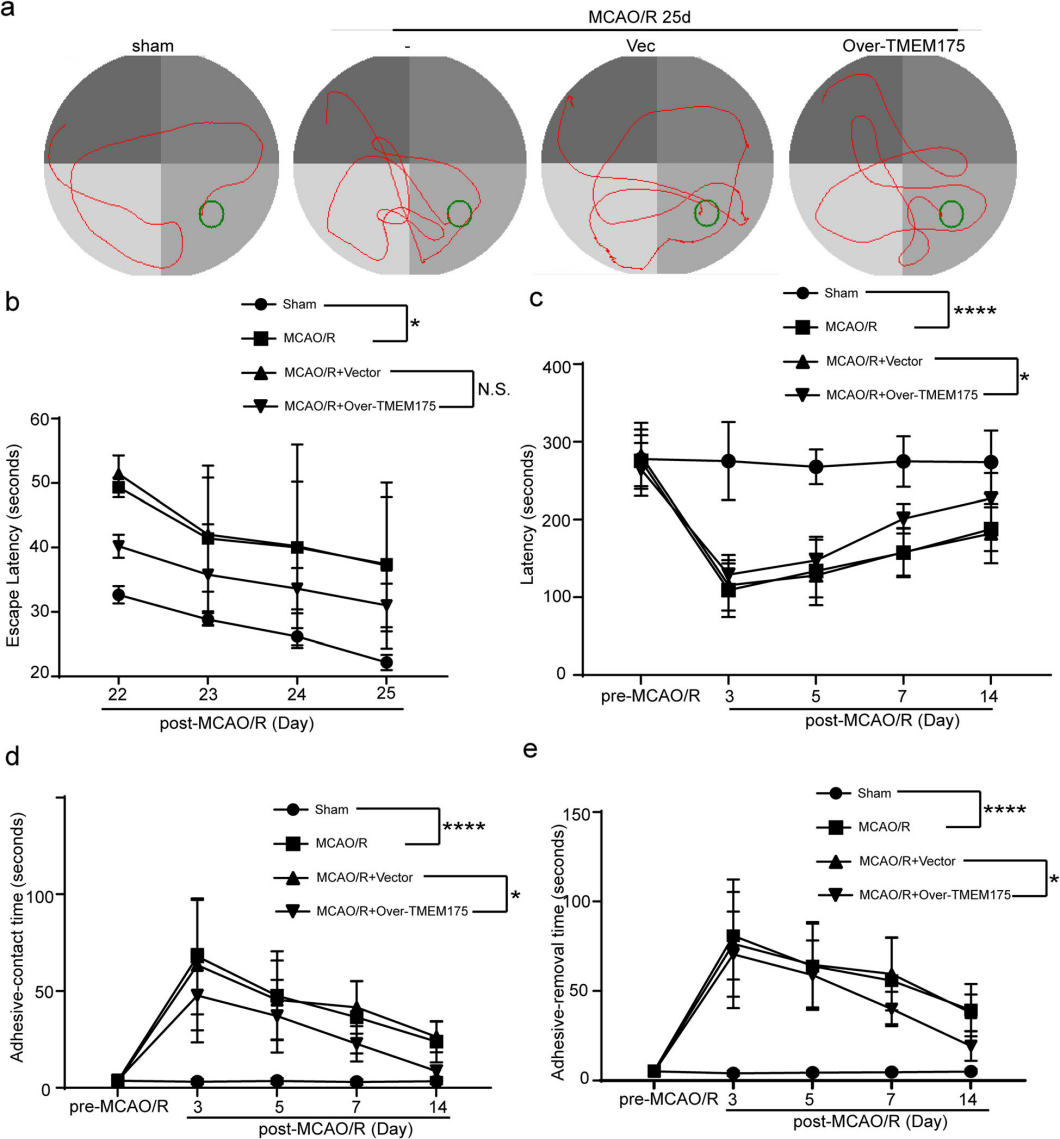
Effects of TMEM175 overexpression on long-term recovery of neurological function after MCAO/R in rats. **a** A typical swim path of rats in the Morris water maze at 25 d after MCAO/R. **b** Time to reach the submerged platform in the Morris water maze at 22–25 d after MCAO/R (MCAO/R vs. sham, *p = 0.0012, Day 22; *p = 0.0317, Day 23; *p = 0.0147, Day 24; *p = 0.0059, Day 25; Vector vs. Over-TMEM175, NS, no significant). **c** Rotarod test (MCAO/R vs. sham, ****p < 0.0001, Day 22–25; Vector vs. Over-TMEM175, *p = 0.0359, Day 7; *p = 0.0263, Day 14). **d** Adhesive-contact time (MCAO/R vs. sham, ****p < 0.0001, Day 3, Day5, Day7, Day14; Vector vs. Over-TMEM175, *p = 0.0268, Day 7; *p = 0.0407, Day 14). **e** Adhesive-removal time (MCAO/R vs. sham, ****p < 0.0001, Day 3, Day5, Day7, Day14; Vector vs. Over-TMEM175, *p = 0.0268, Day 7; *p = 0.0407, Day 14)

To examine the role of TMEM175 deficiency in ischemic injury induced by MCAO/R, we regulated the protein levels of TMEM175 using over-expression constructs in brain tissues of rats. First, we performed overexpression using plasmid transfection of TMEM175 in our rat model of MCAO/R. The results of Western blot analysis showed that, compared with that of the sham group, the level of TMEM175 was significantly decreased in the MCAO/R group, while over-expression of TMEM175 increased the level of TMEM175. The protein expression level of TMEM175 is normalized by GAPDH expression. (Fig. 2a). To explore the roles of TMEM175 in neuronal death and degeneration in brain tissues after MCAO/R, we performed TTC and FJC staining. TTC staining showed that TMEM175 overexpression reduced the infarct volume (Fig. 2b). In addition, FJC staining also revealed a rescuing effect of TMEM175 overexpression on neuronal degeneration. (Fig. 2c). In long-term experiments, as shown in Fig. 3a and b, assessments via the Morris water maze revealed that TMEM175 overexpression did not facilitate spatial learning and memory after MCAO/R. However, TMEM175 upregulation alleviated sensory and motor deficits caused by MCAO/R, as suggested by results of the rotarod test (Fig. 3c) and adhesive-removal test (Fig. 3d and e). Collectively, these data support that TMEM175 overexpression improved neuronal death and neurobehavioral deficits after MCAO/R.

### TMEM175 overexpression reduces neuronal injury induced by OGD/R in vitro

**Fig. 4:**
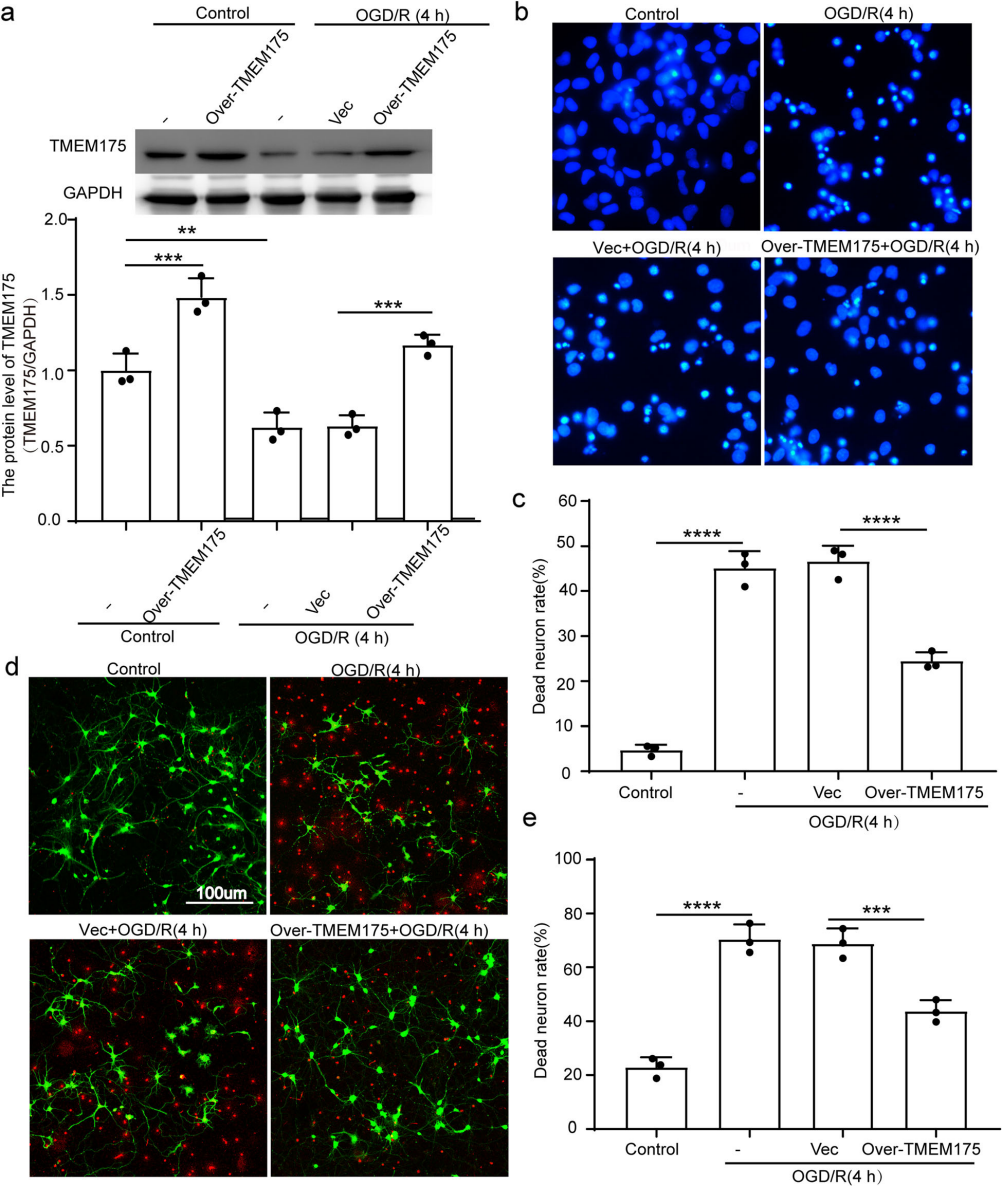
Effects of TMEM175 overexpression on neuronal death after OGD/R. **a** Transfection efficiency of TMEM175 overexpression plasmid in primary neurons (OGD/R vs. control, **p = 0.0054; OGD/R + Vector vs. OGD/R + Over-TMEM175, ***p = 0.0004, control vs. control +Over-TMEM175, ***p = 0.0008). **b** Hoechst staining. The arrow points to dead neurons showing nuclear condensation. **c** Quantitative analysis of cell death via Hoechst staining. Data are presented as the percentage of the dead neurons showing nuclear condensation. (OGD/R vs. control, ****p < 0.0001; Vector vs. Over-TMEM175, ****p < 0.0001). **d** The live/dead cell viability assay. Healthy neurons appear green and the condensed nuclei of dead neurons appear red. **e** Quantitative analysis of cell death via the cell viability live/dead assay. Data are presented as the ratio of dead neurons to live neurons. (OGD/R vs. control, ****p < 0.0001; Vector vs. Over-TMEM175, ***p = 0.0009)

In order to explore the protective role of TMEM175 overexpression under OGD/R conditions, we regulated the protein levels of TMEM175 using over-expression constructs in cultured neurons. To verify the transfection efficiency, we constructed GFP-TMEM175 non-fused overexpressed plasmid. The results showed that the transfection efficiency was stable at about 65% (Supplementary Figure 2). In order to avoid the effect of GFP on the fluorescence detection of Cell-Death/Viability Assays and FJC Staining, our subsequent experiments were carried out based on the transfection of TMEM175 overexpressed plasmid. The results of Western blot analysis showed that, compared with that of the control group, the level of TMEM175 was significantly decreased in the OGD/R group, while over-expression of TMEM175 increased the level of TMEM175. The protein expression level of TMEM175 is normalized by GAPDH expression. (Fig. 4a). Furthermore, we challenged neuronal cultures for 120 min with OGD/R, which is an in-vitro model of ischemia. Four hours later, neuronal death was assessed via Hoechst-33,258 nuclear staining and a live/dead viability assay. As shown in Fig. 4b-e, TMEM175 overexpression reduced the percentage of dead neurons after OGD/R. This result indicates that TMEM175 overexpression increased neuronal resistance against ischemic injury induced by OGD/R.

### TMEM175 Upregulation increased the clearance of damaged mitochondria after OGD/R

**Fig. 5:**
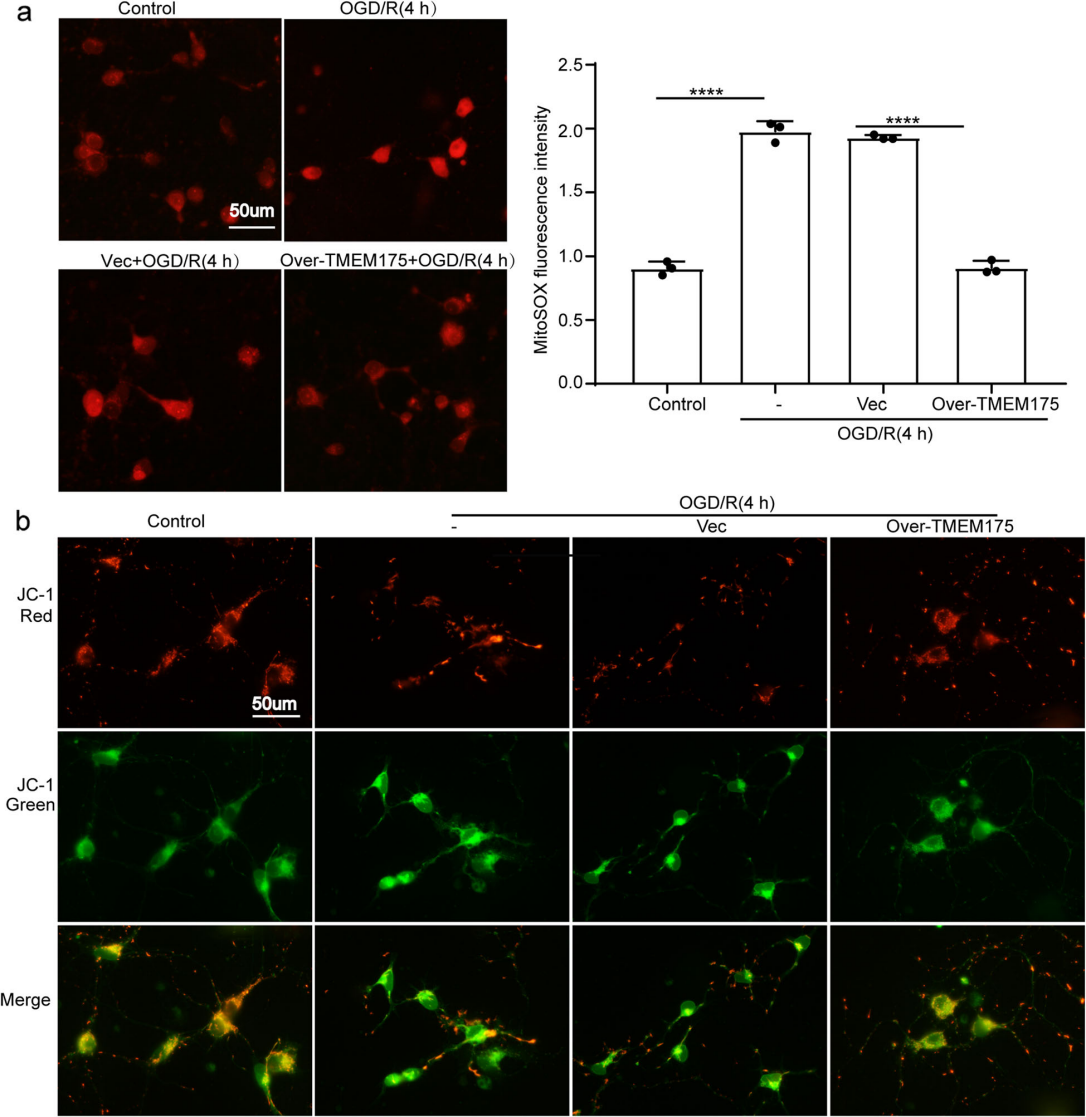
TMEM175 overexpression improves OGD/R-induced impaired mitochondrial quality control in neurons. **a** Detection of the levels of mitochondrial ROS using mito-SOX Red staining. Data are presented as the fluorescence intensity of mito-SOX dye. (OGD/R vs. control, ****p < 0.0001; Vector vs. Over-TMEM175, ****p < 0.0001). **b** Representative images of JC-1 staining from different treatments

We used Mito-Sox staining to detect ROS production under different conditions to measure mitochondrial ROS. As shown in Fig. 5a, ROS levels were significantly increased in the OGD/R group compared with those in the control group. Moreover, TMEM175 over-expression dramatically reduced ROS generation.

Active mitochondria produce red-shifted fluorescence by readily accumulating JC1 dye in mitochondria compared with that of less active mitochondria that instead emit green fluorescence; this ratio of red/green fluorescence is indicative of mitochondrial activity within a given cell. As shown in Fig. 5b, OGD/R treatment significantly reduced mitochondrial activity compared with that of the control group, as evidenced by the high ratio of red/green JC1 florescence. However, TMEM175 overexpression significantly increased mitochondrial activity following OGD/R, as evidenced by a low red/green JC1 fluorescent ratio compared with that of empty-vector-transfected neurons following OGD/R-treated, indicating a protective role of TMEM175 overexpression. Collectively, these results suggest that mitochondria were damaged in the course of ischemic injury and that TMEM175 upregulation ameliorated this damage.

### TMEM175 deficiency induced by OGD/R decreases Lysosomal catalytic activity in neurons via destabilization of Lysosomal pH

**Fig. 6:**
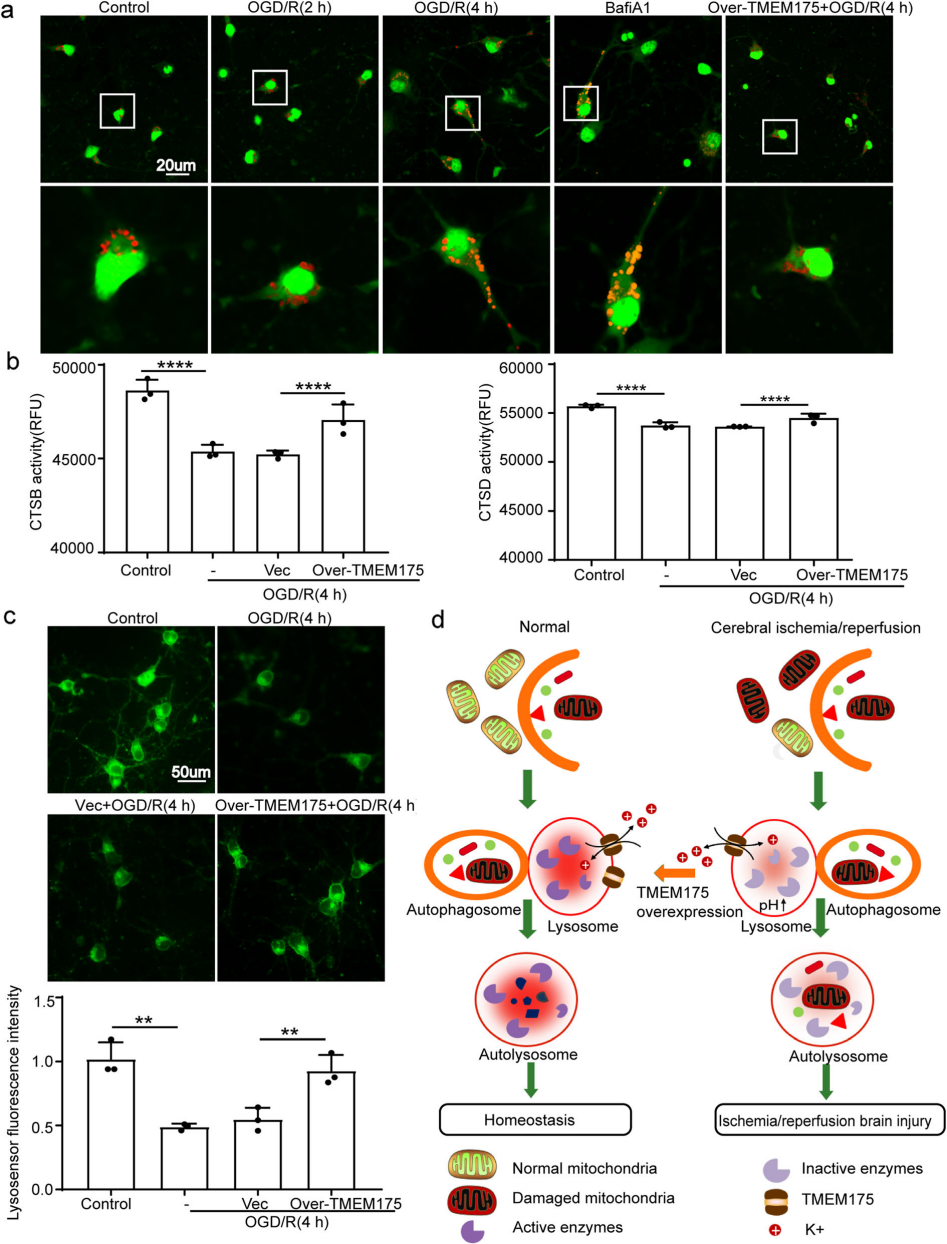
TMEM175 overexpression reverses lysosomal function in neurons exposed to OGD/R. **a** Fluorescent photographs of acridine-orange (AO) staining of neuronal cultures treated with OGD/R, bafilomycin A1 (100nM), or transfection of TMEM175-overexpression plasmid. **b** Relative enzymatic activity of CTSB and CTSD in neurons (OGD/R vs. control, ****p < 0.0001; Vector vs. Over-TMEM175, ****p < 0.0001). **c** Fluorescent photographs of LysoSensor Green staining of neuronal cultures (OGD/R vs. control, **p = 0.001; Vector vs. Over-TMEM175, **p = 0.0082). **d** Schematic representation of potential mechanisms of TMEM175 actions in cerebral I/R injury

TMEM175 is a K^+^ channel that mediates potassium conductance across lysosomal and endosomal membranes and thus regulates lysosomal membrane potential and pH. We evaluated whether TMEM175 deficiency disrupts autophagic flux by inhibiting lysosomal acidification. AO emits red fluorescence in acidic compartments. It is well recognized that bafilomycin A1 disrupts autophagic flux by inhibiting lysosomal acidification; thus, we used it as a positive control. At 4 h after OGD/R in DMEM medium, a significant increase in lysosomal pH was observed. However, compared with that of the bafilomycin A1-treated group, cells after starvation exhibited more bright red fluorescence in the cytoplasm, suggesting that OGD/R affected lysosomal pH (Fig. 6a). To further investigate whether OGD/R affects the hydrolytic function of lysosomes, we measured lysosomal enzymatic activity. As the major lysosomal proteases, the enzymatic activities of CTSB and CTSD were measured. Both CTSB and CTSD activities decreased after OGD/R (Fig. 6b). These results were confirmed by LysoSensor Green staining (Fig. 6c). LysoSensor dyes are acidotropic probes that appear to accumulate in acidic organelles. Thus, LysoSensor reagents exhibit a pH-dependent increase in fluorescent intensity upon acidification. As shown in Fig. 6c, compared with that in the control group, the fluorescent intensity was significantly attenuated in the OGD/R group, which was reversed via TMEM175 overexpression. These results suggest that OGD/R impaired the hydrolytic function and/or quantity of lysosomes.

## Discussion

In this study, we demonstrated that cerebral I/R injury down-regulates the protein level of TMEM175 in neurons. TMEM175 deficiency leads to compromised stability of lysosomal pH, affecting the hydrolytic function of lysosomes. Hence, the process by which dysfunctional proteins and organelles, including damaged mitochondria, are degraded by lysosomes is suppressed. Exogenous up-regulation of TMEM175 protein level could reverse the neuronal lysosomal dysfunction after I/R (Fig. 6d).

Cerebral ischemia is a devastating neurological event with high morbidity and mortality, in which a considerable number of mitochondria are damaged following cerebral I/R injury [22]. Recent evidence has demonstrated that autophagy is a lysosome-regulated process for degrading and recycling cellular constituents and is a gate-keeping mechanism for stabilizing cellular homeostasis [23–25]. Mitophagy is a form of selective autophagy that removes damaged mitochondria for mitochondrial quality control in order to maintain homeostasis [25]. Dysfunctional mitophagy results in insufficient removal of damaged mitochondria [26]. It is noteworthy that the impairment of lysosomal catalytic activity results in a decreased capacity of mitophagy [27, 28]. TMEM175 is a K^+^ channel located in late endosomes and lysosomes. Depletion of TMEM175 alters lysosomal pH and decreased the catalytic capacity of lysosomes [10]. Previous reports have established genetic and physiological evidence that TMEM175 is a potential risk factor and candidate therapeutic target for Parkinson’s disease and Alzheimer’s disease [7, 11]. However, whether TMEM175 also participates in regulating brain-injury-induced mitophagy has remained unclear.

In the present study, we found that the level of TMEM175 in brain tissues was decreased after ischemic injury, as determined by Western blotting. Analysis of the time course of TMEM175 levels in brain tissues after MCAO/R revealed that TMEM175 levels were significantly decreased starting at 3 h and rebounded at 12 h after MCAO/R (Fig. 1a). MicroRNAs (miRNAs) are a class of non-coding RNA molecules about 22 nucleotides in size that are involved in the regulation of gene expression at the post-transcriptional level. It is estimated that about 30% of human gene transcriptome are direct targets of miRNAs, and nearly 90% of human genes are regulated by miRNAs [29]. Compared with the regulation of gene expression at the transcriptional level, the regulation of gene expression at the post-transcriptional level is more rapid, and the latter may be more suitable for CNS cells to deal with acute stress such as stroke. Based on targetscan prediction, we found that hsa-miR-1268a, hsa-miR-1268band hsa-miR-585-3p have the potential to target TMEM175 mRNA 3’UTR. Studies have shown that hsa-miR-1268a, hsa-miR-1268b and hsa-miR-585-3p levels change in cardiovascular diseases and tumors [30–32], suggesting that their levels are regulated by pathological environment. Therefore, we speculated that the loss of TMEM175 induced by I/R might be regulated by miRNAs such as hsa-miR-1268a, hsa-miR-1268b and hsa-miR-585-3p, which will be further verified in our follow-up work.

Subsequently, we used TMEM175-overexpression plasmids to increase TMEM175 levels in rats prior to the induction of MCAO/R. We found that TMEM175 protein levels in brain tissue were increased at 24 h after MCAO/R and were negatively correlated with the expression of TMEM175 (Fig. 2a). Additionally, we also found that neuronal necrosis in the TMEM175-overexpression group was significantly improved compared with that of the control group, and neurobehavioral tests yielded consistent results (Fig. 2 and 3). Therefore, we conclude that TMEM175 deficiency exerts a negative effect on brain tissue in terms of short-time and long-term brain injury after MCAO/R. Furthermore, TMEM175 deficiency exhibited the same effects in vitro (Fig. 4). We found that damage to neurons induced increased mitochondrial-derived ROS levels, which was reversed by TMEM175 overexpression (Fig. 5a). We also explored the underlying mechanism of TMEM175 deficiency on insufficient removal of damaged mitochondria. The data from acidophilic-dye staining suggested that OGD/R-injury induced unstable lysosomal pH in primary neurons (Fig. 6a and c).

Existing data points to the relationship between lysosomal pH and the viability of cells. Bafilomycin A1 is a commonly used compound that inhibits autophagic flux by preventing the acidification of endosomes and lysosomes [33, 34]. In addition, a previous study has shown that Bafilomycin A1 decreased cell viability [26]. Therefore, we could conclude that the pH change exerted an effect on cell viability of injured cells.

CTSB and CTSD are the most abundant lysosomal cysteine and aspartyl proteases that contribute to the degradation of autolysosomes [35]. The enzymatic function of these enzymes is influenced by the pH of the surrounding environment. In our present study, the enzymatic activities of CTSB and CTSD were also altered after OGD/R treatment (Fig. 6b), suggesting that TMEM175 deficiency exerts a negative effect on the hydrolytic function of lysosomes following cerebral ischemia with reperfusion. Hence, TMEM175 deficiency may represent a novel late-stage mitophagic inhibitor. Emerging data indicate that mitophagic activation may hold promise as a potential therapeutic strategy against ischemic brain injury [36]. Nevertheless, the mechanisms underlying mitophagy in ischemic neurons are not fully understood. Recent investigations have highlighted roles of PARK2, an E3 ubiquitin ligase, as well as BNIP3L/NIX, in mitophagy [37–42]. TMEM175 is a novel K^+^ channel independent of these proteins. It is noteworthy that impaired mitophagy might link TMEM175 deficiency to impairment of mitochondrial clearance. Direct inhibition of autophagy through genetic and pharmacological methods has been shown to decrease mitochondrial oxygen consumption and energy production through the accumulation of dysfunctional mitochondria [43–47]. TMEM175-deficient cells may also be caused by these mechanisms. However, the exact mechanism remains to be elucidated.

Several limitations of our present study should be noted. First, we demonstrated that TMEM175 deficiency inhibited lysosomal function via unstable pH and decreased protease activity. However, whether the integrity of the lysosome is also compromised by the dysregulation of lysosomal pH requires further investigation. Additionally, although our current study provides evidence that TMEM175 deficiency may disrupt autophagic flux by inhibiting lysosomal acidification, we did not more deeply address whether TMEM175 deficiency affects the fusion of autophagosomes with lysosomes after OGD/R. Second, since we only detected TMEM175 expression within 48 h after MCAO/R, TMEM175 expression levels in chronic-stage post- MCAO/R remain unclear. Third, we only interfered with the expression of TMEM175 using a TMEM175-overexpression plasmid. To reduce the level of TMEM175 expression, a specific siRNA of TMEM175 or knockout of TMEM175 (KO) should be applied in future studies to determine the effect of TMEM175 depletion in I/R injury. Finally, only adult male SD rats are used in our study.

In summary, our present study represents the first to investigate the expression of TMEM175 in MCAO/R rats, and we found that TMEM175 deficiency may be harmful to the recovery of ischemic injury. Furthermore, we found that TMEM175 is associated with lysosomal pH, lysosomal proteolytic activity, and mitochondrial clearance. Therefore, this study lays the foundation for further investigations on the regulatory mechanisms of TMEM175 in the course of autophagy in I/R injury and suggests that TMEM175 may represent a therapeutic target for the amelioration of brain injury after I/R injury.

## Supplementary information

**Additional file 1.** Supplementary material.

## Data Availability

The datasets generated and/or analysed during the current study are not publicly available due to the confidential policy of our hospital but are available from the corresponding author on reasonable request.

## Abbreviations

I/R: Ischemia-reperfusion
MCAO/R: Middle-cerebral-artery occlusion/reperfusion
OGD/R: Oxygen-glucose deprivation/reoxygenation
AO staining: Acridine orange staining
CTSB: Cathepsin-B
CTSD: Cathepsin-D
FJC staining: Fluoro-Jade C (FJC) staining
TTC staining: 2,3,5-Triphenyltetrazolium Chloride staining
ROS: Reactive Oxygen Species
