# Supplementary material for: TMEM175 mediates Lysosomal function and participates in neuronal injury induced by cerebral ischemia-reperfusion
Source: Mol Brain. 2020 Aug 15;13:113. doi: 10.1186/s13041-020-00651-z (PMC7429711; doi:10.1186/s13041-020-00651-z)
Supplement: Supplementary file 1 — Additional file 1. Supplementary material. [file 13041_2020_651_MOESM1_ESM.docx]

**TMEM175 Mediates Lysosomal Function and Participates in Neuronal Injury Induced by Cerebral Ischemia-Reperfusion**

**Supplementary Figures and Figure legends**

Supplementary Figure 1


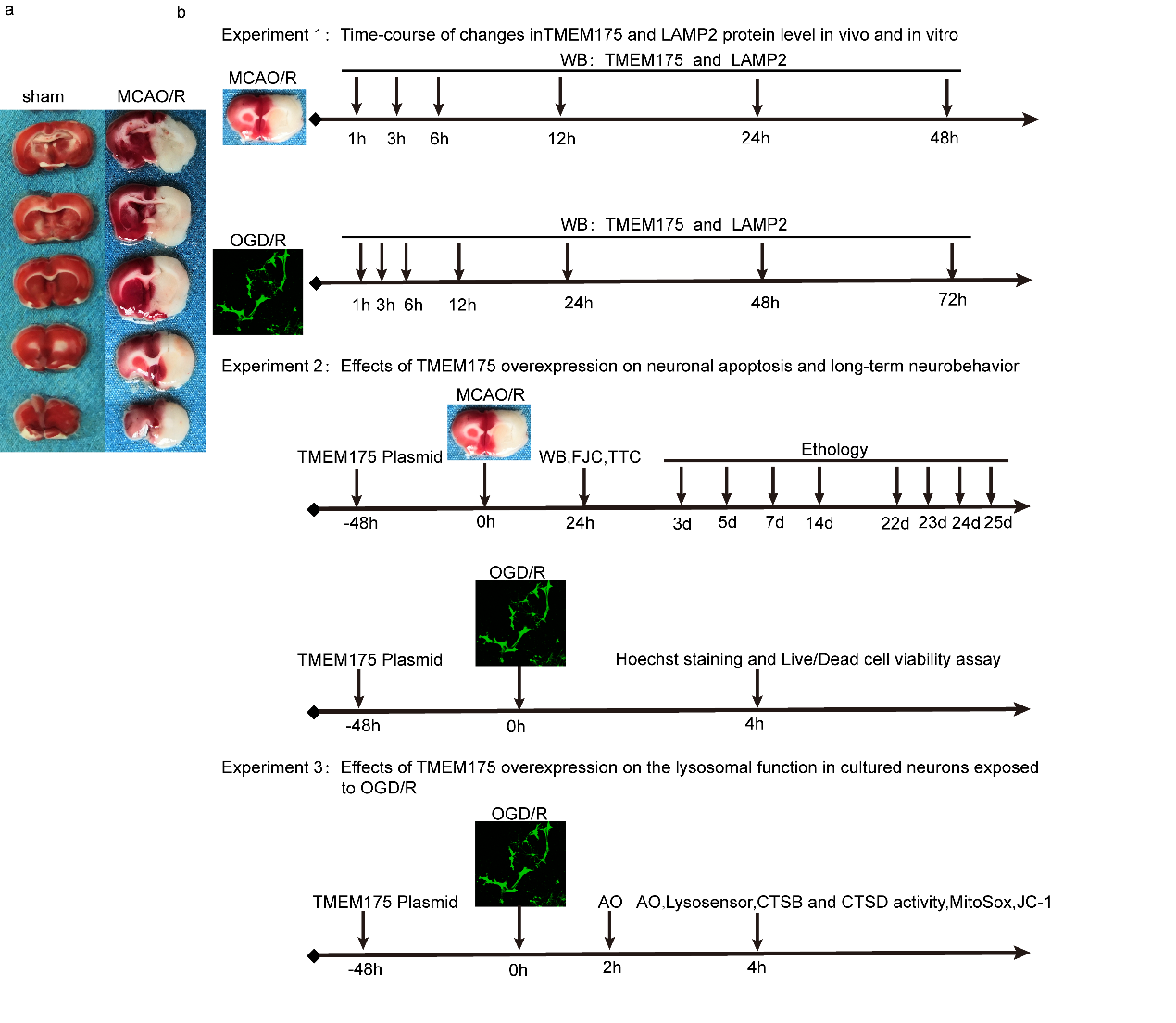


Supplementary Figure 1. Establishment of MCAO/R model and experimental design. (a) The rat MCAO model was evaluated by TTC staining. (b) Experiment 1: Changes in TMEM175 and LAMP2 protein levels at diﬀerent time points after MCAO/R and OGD/R. Experiment 2: Effects of TMEM175 overexpression on neuronal apoptosis and long-term behavior. Experiment 3: Effects of TMEM175 overexpression on the lysosomal function in cultured neurons exposed to OGD/R.

Supplementary Figure 2


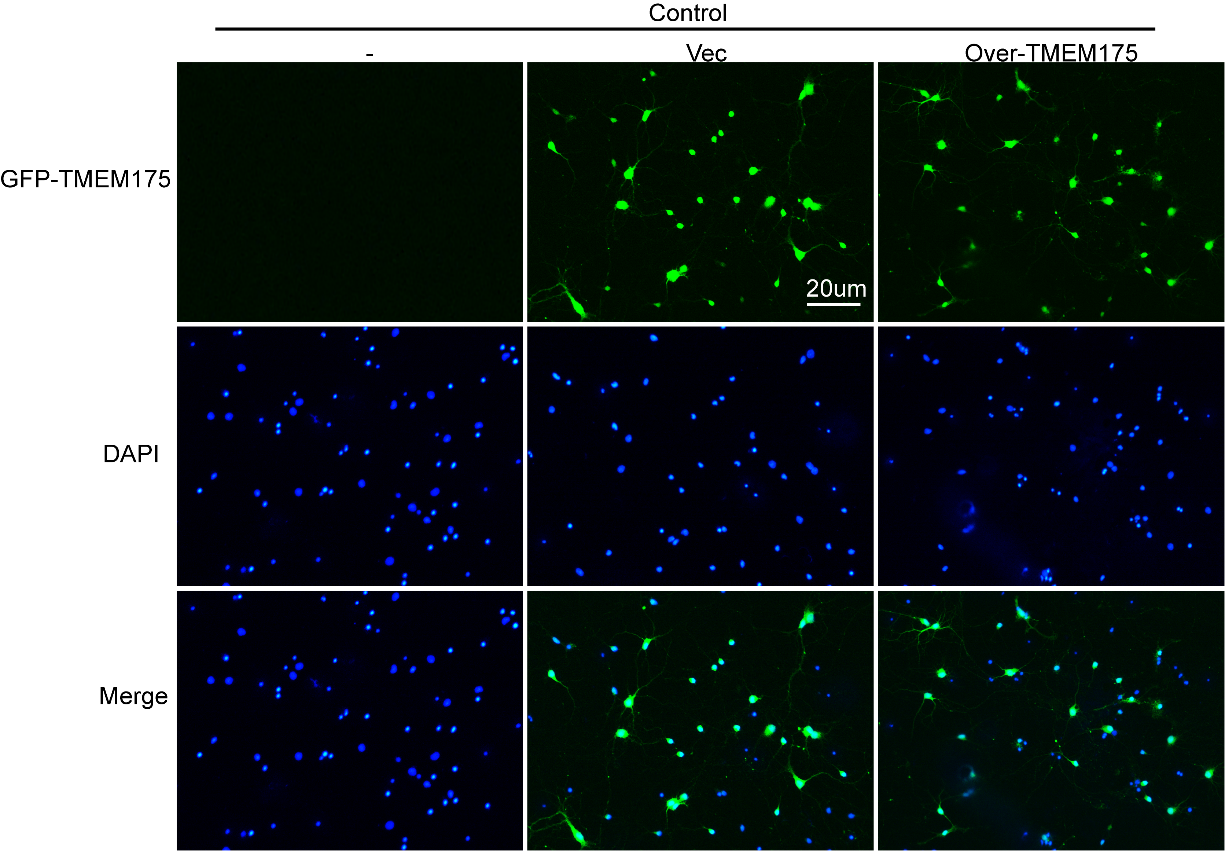


Supplementary Figure 2. Transfection eﬃciency of the GFP-TMEM175 non-fused overexpressed plasmid.

**Supplementary Tables**

Supplementary Table 1. Statistical table

|  | Description | In vivo or in vitro | Test used | Stat-value | One or two tailed P value |
| --- | --- | --- | --- | --- | --- |
| Fig.1a | The protein level of TMEM175 | In vivo | One-way ANOVA | F=7.687, P=0.0001, 95%CI=0.2044 to 0.8921 (sham vs. MCAO/R12 h)  P=0.0248,95%CI=-0.7194 to -0.03169(MCAO/R12 h vs. MCAO/R48h)  η2=0.5684 | Two-tailed |
| Fig.1b | The protein level of TMEM175 | In vitro | One-way ANOVA | F=2.209, P=0.0457, 95%CI=-1.102 to -0.008735(control vs. OGD/R3h).  P=0.0192,95%CI=  -1.183 to -0.0904  (OGD/R12 h vs. OGD/R48h)  η2=0.4916 | Two-tailed |
| Fig.2a | The protein level of TMEM175 | In vivo | One-way ANOVA | F=17.55, P=0.0215  , 95%CI=  0.05849 to 0.9716  (sham vs. MCAO/R). P=0.0091,95%CI=  -1.028 to -0.1147  (MCAO/R+Vector vs.MCAO/R+Over-TMEM175). P=0.0034,95%CI=  -1.09 to -0.1771  (sham vs. sham + Over-TMEM175).  η2=0.7375 | Two-tailed |
| Fig.2b | Infarct volume/total volume | In vivo | One-way ANOVA | F=8.146, P=0.0126  , 95%CI=0.02545 to 0.2124(MCAO/R+Vector vs.MCAO/R+Over-TMEM175). η2=0.5206 | Two-tailed |
| Fig.2c | FJC positive cells | In vivo | One-way ANOVA | F=61.22, P=0.0001, 95%CI=16.7 to 29.3  (MCAO/R+Vector vs. MCAO/R+Over-TMEM175). η2=0.8909 | Two-tailed |
| Fig.3b | Escape latency | In vivo | Two-way ANOVA | Not significant (MCAO/R+Vector vs. MCAO/R+Over-TMEM175) | Two-tailed |
| Fig.3c | Latency | In vivo | Two-way ANOVA | F = 8.254, P=0.0359  , 95%CI=-83.76 to -2.236  (MCAO/R+Vector vs. MCAO/R+Over-TMEM175, Day7).  P=0.0263, 95%CI=  -85.76 to -4.236  (MCAO/R+Vector vs. MCAO/R+Over-TMEM175, Day14).  η2=0.9894 | Two-tailed |
| Fig.3d | Adhesive-contact time | In vivo | Two-way ANOVA | F= 5.908, P=0.0268  , 95%CI=1.728 to 35.94  (MCAO/R+Vector vs. MCAO/R+Over-TMEM175, Day7).  P=0.0407, 95%CI=  0.5891 to 34.8  (MCAO/R+Vector vs. MCAO/R+Over-TMEM175, Day14).  η2=0.4819 | Two-tailed |
| Fig.3e | Adhesive-removal time | In vivo | Two-way ANOVA | F= 8.328, P=0.0322  , 95%CI=1.321 to 37.9  (MCAO/R+Vector vs. MCAO/R+Over-TMEM175, Day7).  P=0.0463, 95%CI=  0.2379 to 36.82  (MCAO/R+Vector vs. MCAO/R+Over-TMEM175, Day14).  η2=0.6048 | Two-tailed |
| Fig.4a | The protein level of TMEM175 | In vitro | One-way ANOVA | F=43.08, P=0.0054  , 95%CI=0.1164 to 0.6388  (control vs. OGD/R).  P=0.0004,95%CI=  -0.7993 to -0.2769  OGD/R+Vector vs.OGD/R+Over-TMEM175. P=0.0008  , 95%CI= -0.7482 to -0.2258  (control vs. control+Over-TMEM175).  η2=0.9399 | Two-tailed |
| Fig.4c | Dead neuron rate | In vitro | One-way ANOVA | F=148.2, P=0.0001  , 95%CI=  -0.4773 to -0.3303  (control vs. OGD/R). P=0.0001,95%CI=  0.1476 to 0.2946  (OGD/R+Vector vs.OGD/R+Over-TMEM175)  η2=0.9824 | Two-tailed |
| Fig.4e | Dead neuron rate | In vitro | One-way ANOVA | F=67, P=0.0001  , 95%CI=  -0.6004 to -0.3497  (control vs. OGD/R).  P=0.0009,95%CI=  0.1268 to 0.3775  (OGD/R+Vector vs.OGD/R+Over-TMEM175)  η2=0.9617 | Two-tailed |
| Fig.5a | Mito-SOX fluorescence intensity | In vitro | One-way ANOVA | F=360.5, P=0.0001  , 95%CI=-1.219 to -0.9301  (control vs. OGD/R).  P=0.0001,95%CI=0.8762 to 1.165  (OGD/R+Vector vs.OGD/R+Over-TMEM175)  η2=0.9925 | Two-tailed |
| Fig.6b | CTSB activity | In vitro | One-way ANOVA | F=25.54, P=0.0004  , 95%CI=-1816 to 4689  (control vs. OGD/R).  P=0.0149,95%CI=  -3267 to -394.2  (OGD/R+Vector vs.OGD/R+Over-TMEM175)  η2=0.9005 | Two-tailed |
| Fig.6b | CTSD activity | In vitro | One-way ANOVA | F=32.19, P=0.0002  , 95%CI=1198 to 2724  (control vs. OGD/R).  P=0.0262,95%CI=  -1636 to -110.5  (OGD/R+Vector vs.OGD/R+Over-TMEM175)  η2=0.9235 | One-way ANOVA |
| Fig.6c | Lysosensor  fluorescence intensity | In vitro | One-way ANOVA | F=20.2, P=0.0010  , 95%CI=0.2609 to 0.7978  (control vs. OGD/R).  P=0.0082,95%CI=  -0.6495 to -0.1126  (OGD/R+Vector vs.OGD/R+Over-TMEM175)  η2=0.8834 | Two-tailed |

Supplementary Table 2. Rat mortality

| Groups | Mortality Rate |
| --- | --- |
| Experiment 1  Sham  MCAO/R (1 h, 3 h, 6 h, 12 h, 24 h, 48 h)  Experiment 2  Sham  Sham + TMEM175 plasmid  MCAO/R  MCAO/R + Vector  MCAO/R + TMEM175 plasmid  Total  Sham  Sham + TMEM175 plasmid  MCAO/R | 0% (0/6)  25% (12/48)  0% (0/34)  5.6% (2/36)  15% (6/40)  12.8% (5/39)  22.7% (10/44)  14.2% (35/247)  0% (0/40)  5.6% (2/36)  19.3% (33/171) |
